# Supplementary material for: A yeast phenomic model for the influence of Warburg metabolism on genetic buffering of doxorubicin
Source: Cancer Metab. 2019 Oct 23;7:9. doi: 10.1186/s40170-019-0201-3 (PMC6806529; doi:10.1186/s40170-019-0201-3)
Supplement: Supplementary file 9 — Additional file 9. GO term-specific heatmaps for REMc/GTF-enriched clusters. GO term-specific heatmaps for significant GO process terms were generated as described in methods and Figs. 3 and 4. Any related child terms are presented in subsequent pages of the parent file name. GO terms with more than 100 children, with 2 or fewer genes annotated to the term, or a file size over 300KB are not shown. All heatmaps are generated with the same layout (see Figs. 3 and 4). [file 40170_2019_201_MOESM9_ESM.bz2 › Additional_File9_GOTermHeatmaps/Additional_File9_GOTermHeatmap/protein_localization_to_nucleus.pdf]

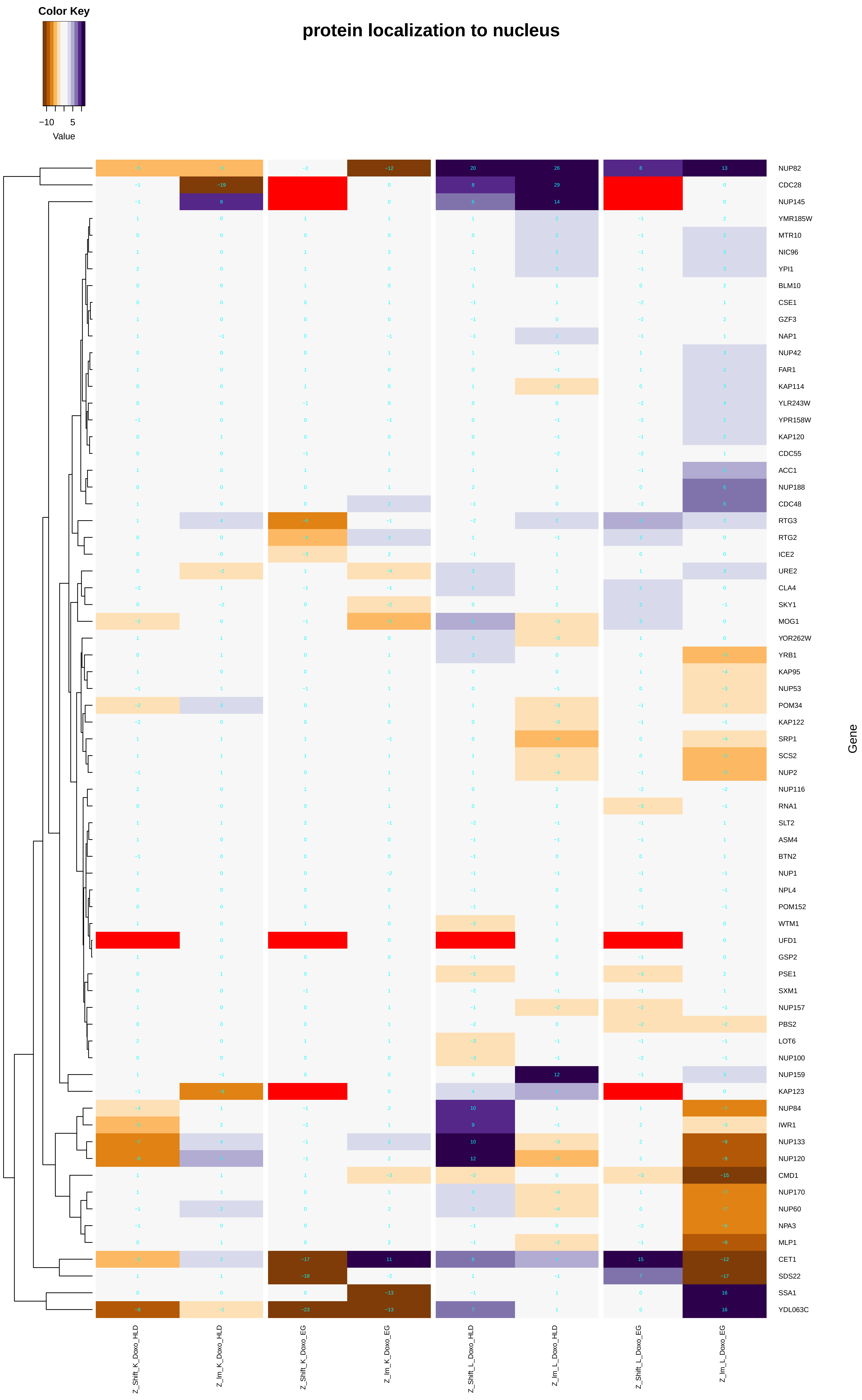
protein localization to nucleus
Color Key
−10
5
Value
Gene
NUP82
CDC28
NUP145
YMR185W
MTR10
NIC96
YPI1
BLM10
CSE1
GZF3
NAP1
NUP42
FAR1
KAP114
YLR243W
YPR158W
KAP120
CDC55
ACC1
NUP188
CDC48
RTG3
RTG2
ICE2
URE2
CLA4
SKY1
MOG1
YOR262W
YRB1
KAP95
NUP53
POM34
KAP122
SRP1
SCS2
NUP2
NUP116
RNA1
SLT2
ASM4
BTN2
NUP1
NPL4
POM152
WTM1
UFD1
GSP2
PSE1
SXM1
NUP157
PBS2
LOT6
NUP100
NUP159
KAP123
NUP84
IWR1
NUP133
NUP120
CMD1
NUP170
NUP60
NPA3
MLP1
CET1
SDS22
SSA1
YDL063C
Z_Shift_K_Doxo_HLD
Z_lm_K_Doxo_HLD
Z_Shift_K_Doxo_EG
Z_lm_K_Doxo_EG
Z_Shift_L_Doxo_HLD
Z_lm_L_Doxo_HLD
Z_Shift_L_Doxo_EG
Z_lm_L_Doxo_EG

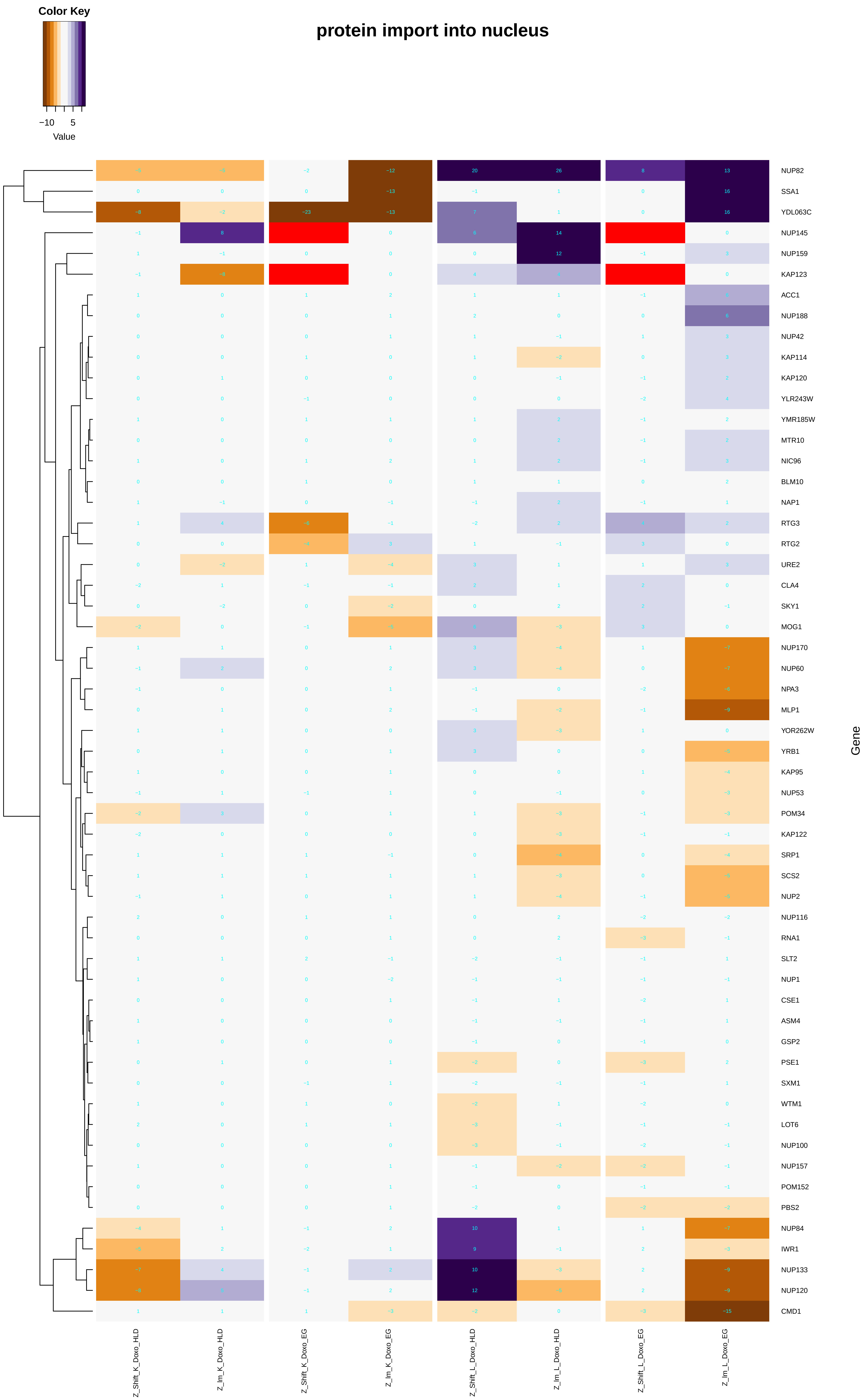
Color Key
−10
5
Value
protein import into nucleus
NUP82
SSA1
YDL063C
NUP145
NUP159
KAP123
ACC1
NUP188
NUP42
KAP114
KAP120
YLR243W
YMR185W
MTR10
NIC96
BLM10
NAP1
RTG3
RTG2
URE2
CLA4
SKY1
MOG1
NUP170
NUP60
NPA3
MLP1
YOR262W
YRB1
KAP95
NUP53
POM34
KAP122
SRP1
SCS2
NUP2
NUP116
RNA1
SLT2
NUP1
CSE1
ASM4
GSP2
PSE1
SXM1
WTM1
LOT6
NUP100
NUP157
POM152
PBS2
NUP84
IWR1
NUP133
NUP120
CMD1
Gene
Z_Shift_K_Doxo_HLD
Z_Im_K_Doxo_HLD
Z_Shift_K_Doxo_EG
Z_Im_K_Doxo_EG
Z_Shift_L_Doxo_HLD
Z_Im_L_Doxo_HLD
Z_Shift_L_Doxo_EG
Z_Im_L_Doxo_EG

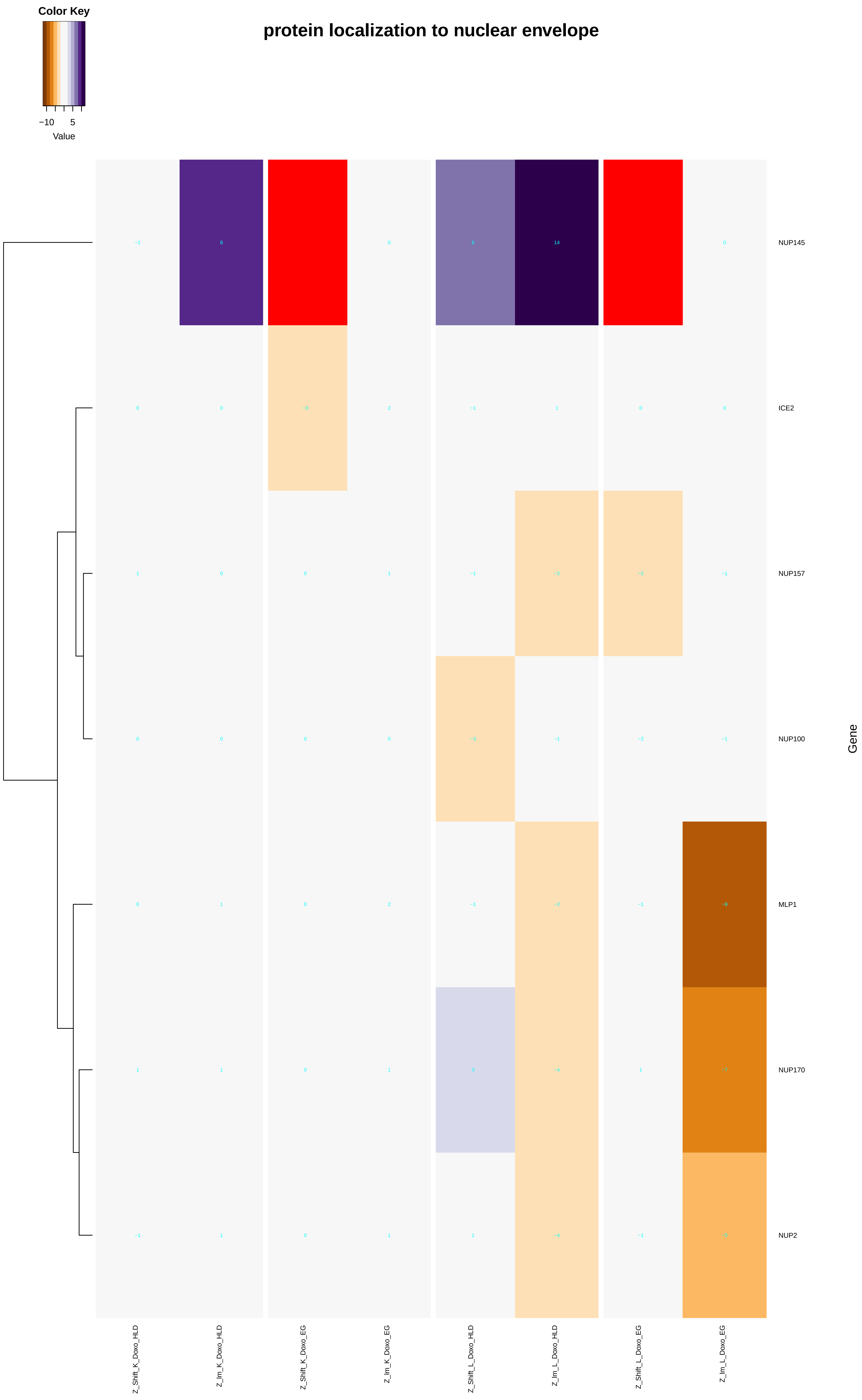

protein localization to nuclear envelope
Color Key
−10
5
Value
NUP145
ICE2
NUP157
NUP100
MLP1
NUP170
NUP2
Gene
Z_Shift_K_Doxo_HLD
Z_lm_K_Doxo_HLD
Z_Shift_K_Doxo_EG
Z_lm_K_Doxo_EG
Z_Shift_L_Doxo_HLD
Z_lm_L_Doxo_HLD
Z_Shift_L_Doxo_EG
Z_lm_L_Doxo_EG

RNA polymerase III complex localization to nucleus

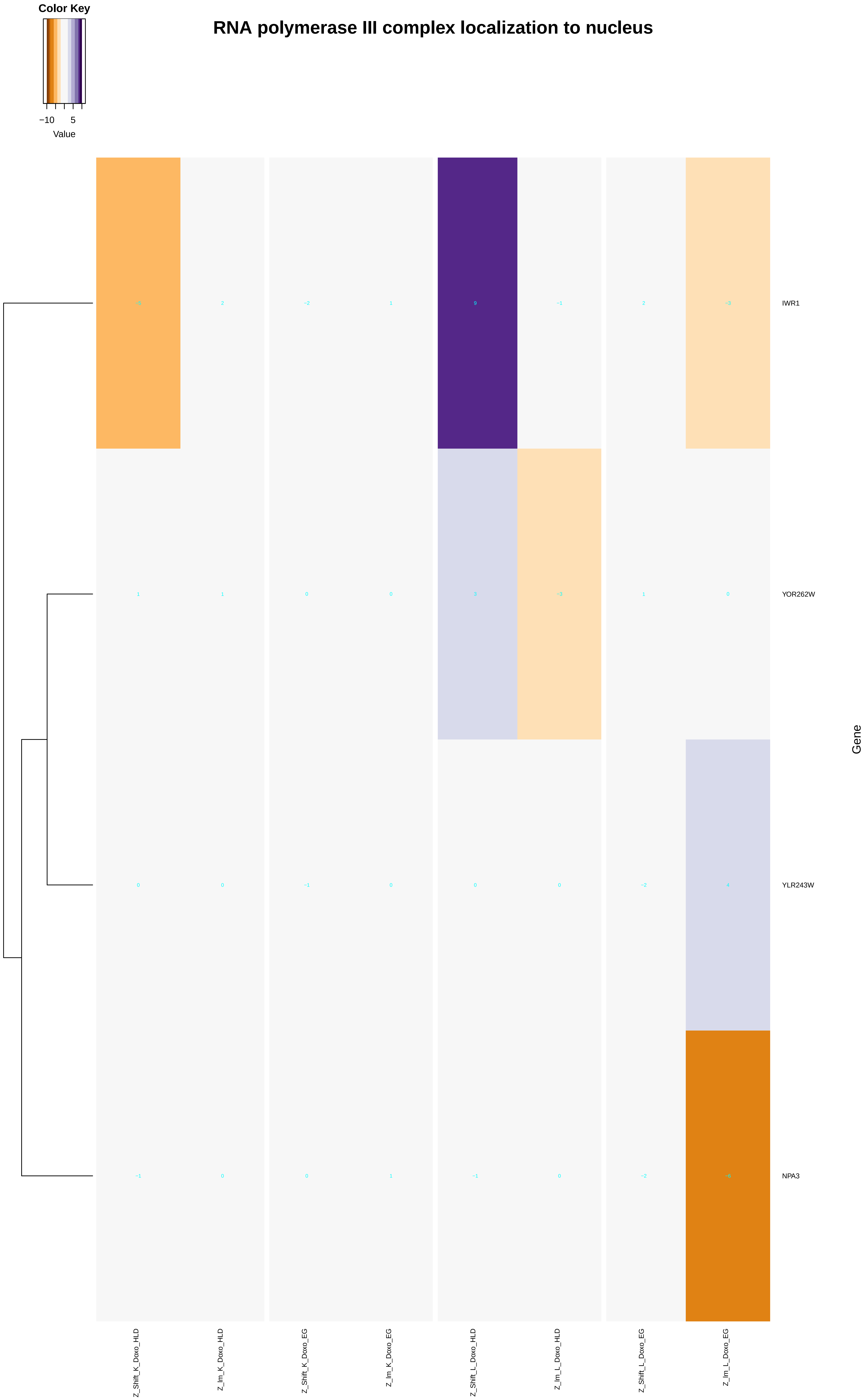

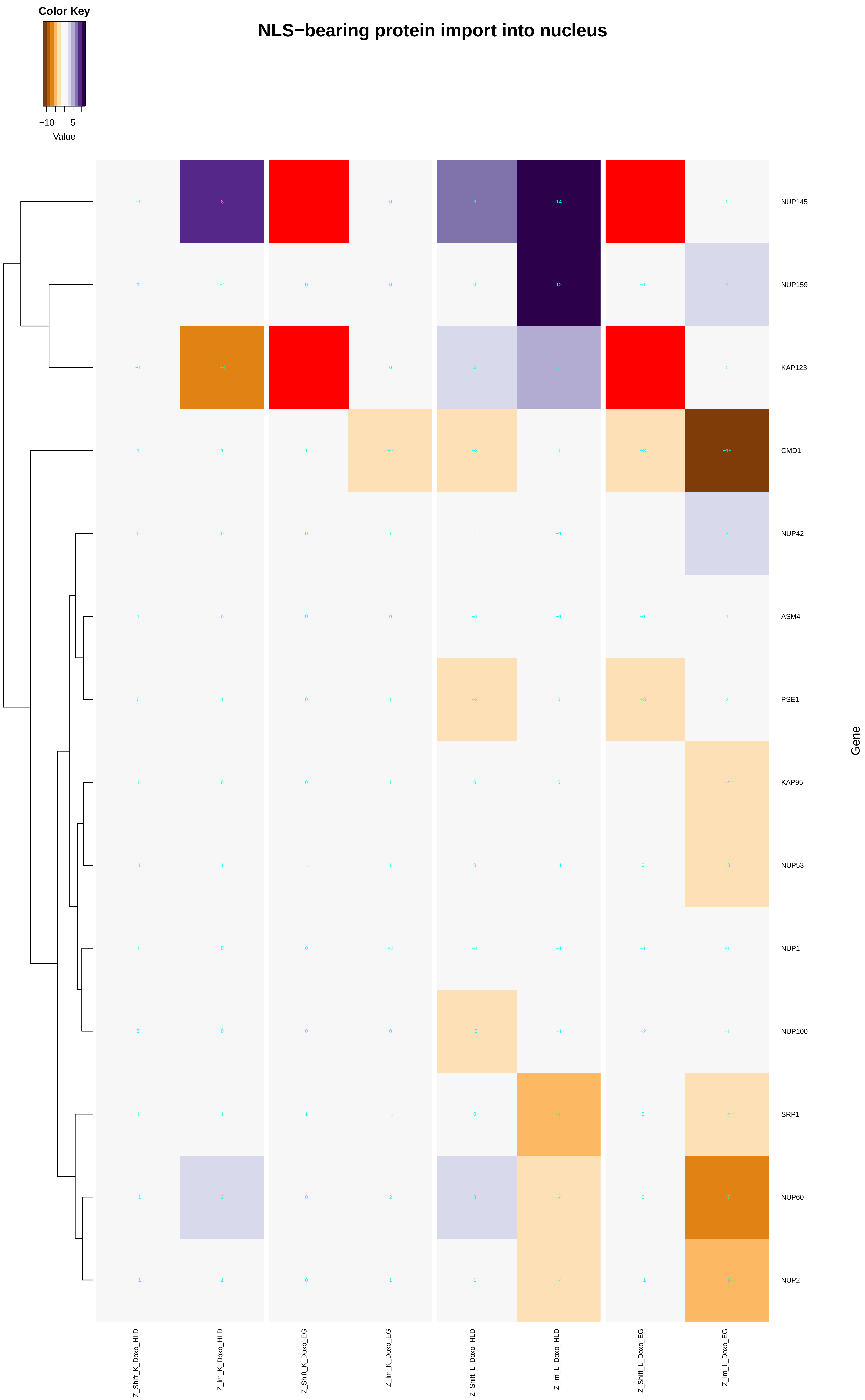
NLS−bearing protein import into nucleus
Color Key
−10
5
Value
NUP145
NUP159
KAP123
CMD1
NUP42
ASM4
PSE1
KAP95
NUP53
NUP1
NUP100
SRP1
NUP60
NUP2
Gene
Z_Shift_K_Doxo_HLD
Z_Im_K_Doxo_HLD
Z_Shift_K_Doxo_EG
Z_Im_K_Doxo_EG
Z_Shift_L_Doxo_HLD
Z_Im_L_Doxo_HLD
Z_Shift_L_Doxo_EG
Z_Im_L_Doxo_EG

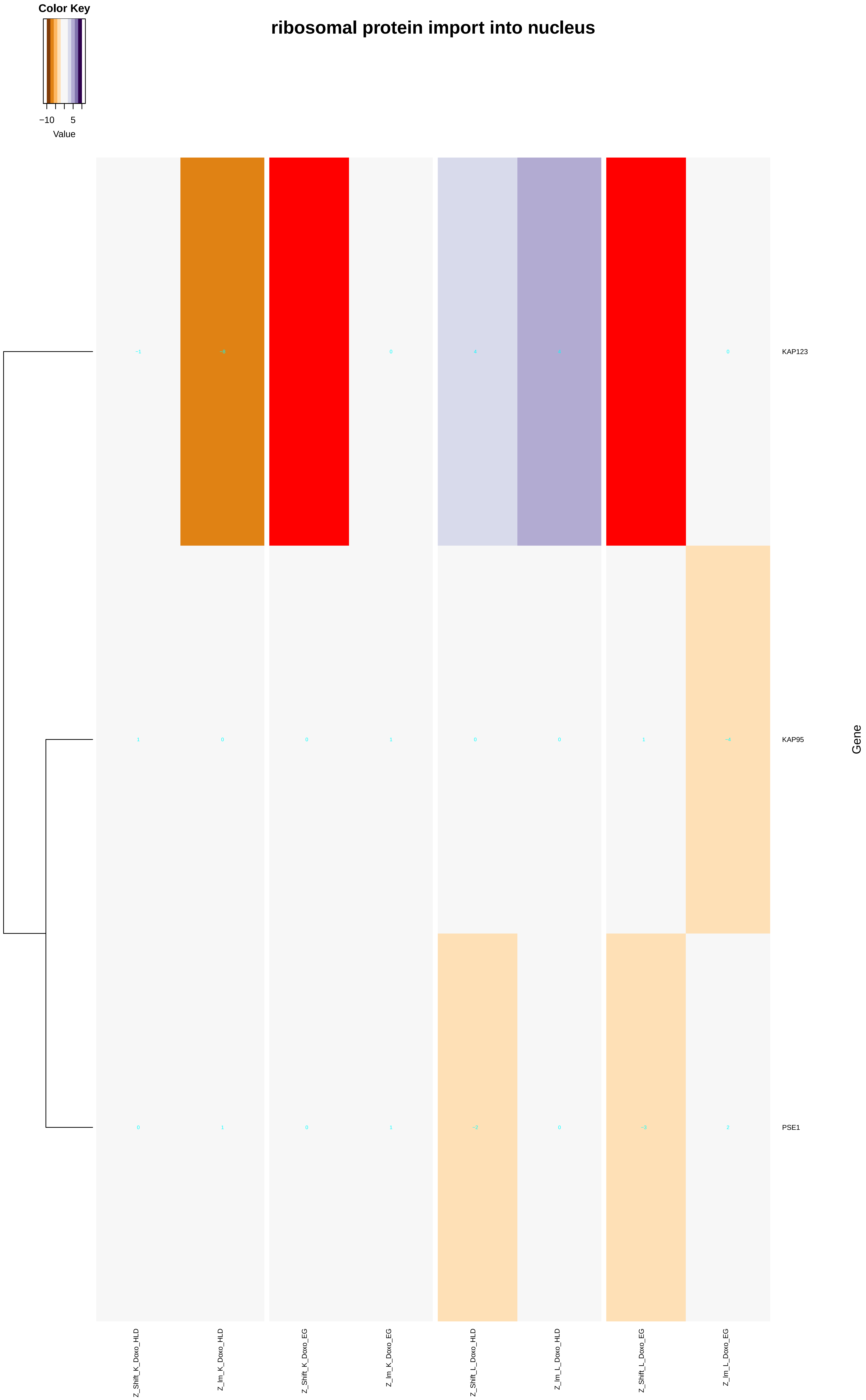

Color Key
−10
5
Value
ribosomal protein import into nucleus
KAP123
KAP95
PSE1
Gene
Z_Shift_K_Doxo_HLD
Z_Im_K_Doxo_HLD
Z_Shift_K_Doxo_EG
Z_Im_K_Doxo_EG
Z_Shift_L_Doxo_HLD
Z_Im_L_Doxo_HLD
Z_Shift_L_Doxo_EG
Z_Im_L_Doxo_EG

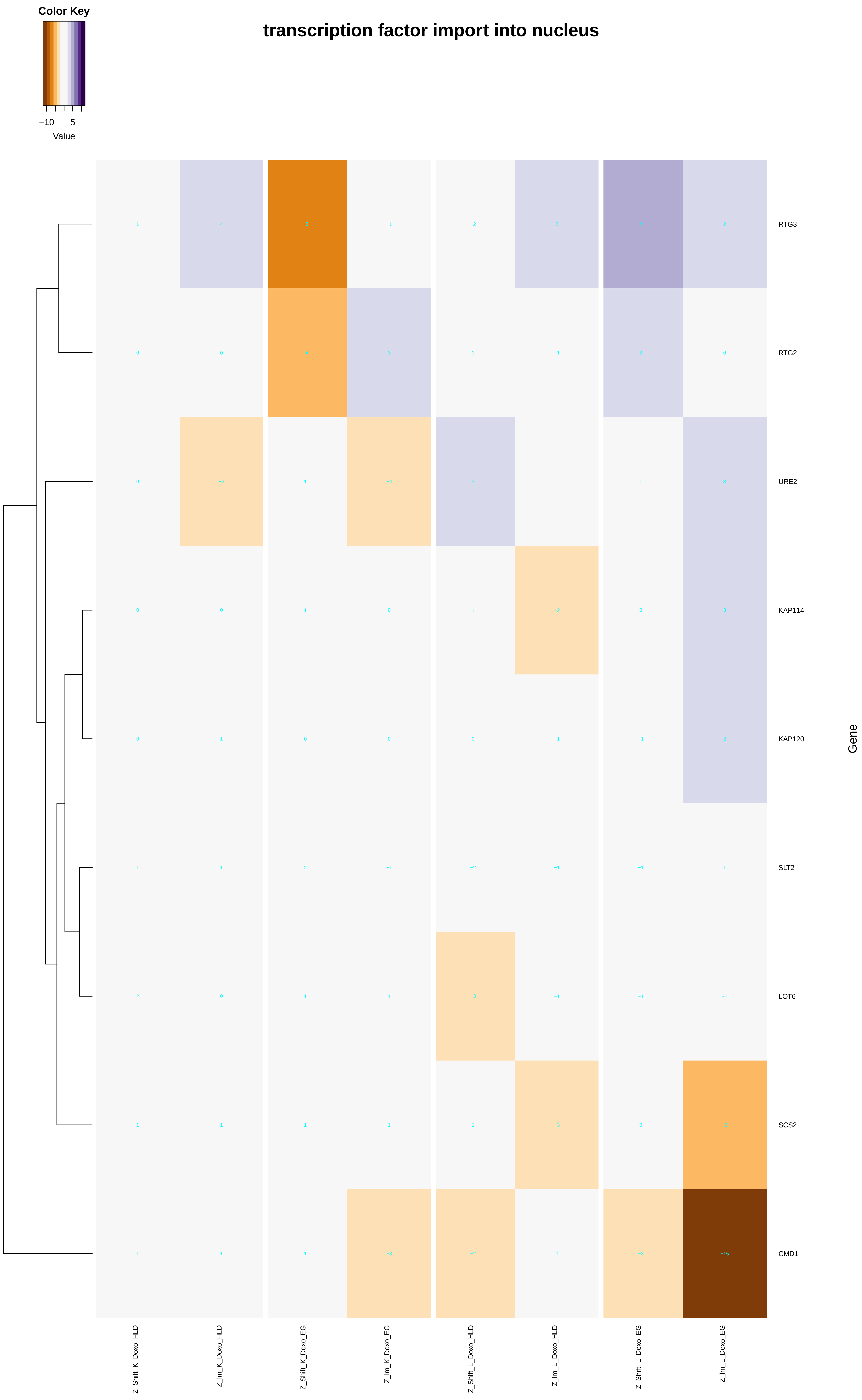
Color Key
−10
5
Value
transcription factor import into nucleus
RTG3
RTG2
URE2
KAP114
KAP120
SLT2
LOT6
SCS2
CMD1
Gene
Z_Shift_K_Doxo_HLD
Z_Im_K_Doxo_HLD
Z_Shift_K_Doxo_EG
Z_Im_K_Doxo_EG
Z_Shift_L_Doxo_HLD
Z_Im_L_Doxo_HLD
Z_Shift_L_Doxo_EG
Z_Im_L_Doxo_EG

RNA polymerase II complex import to nucleus

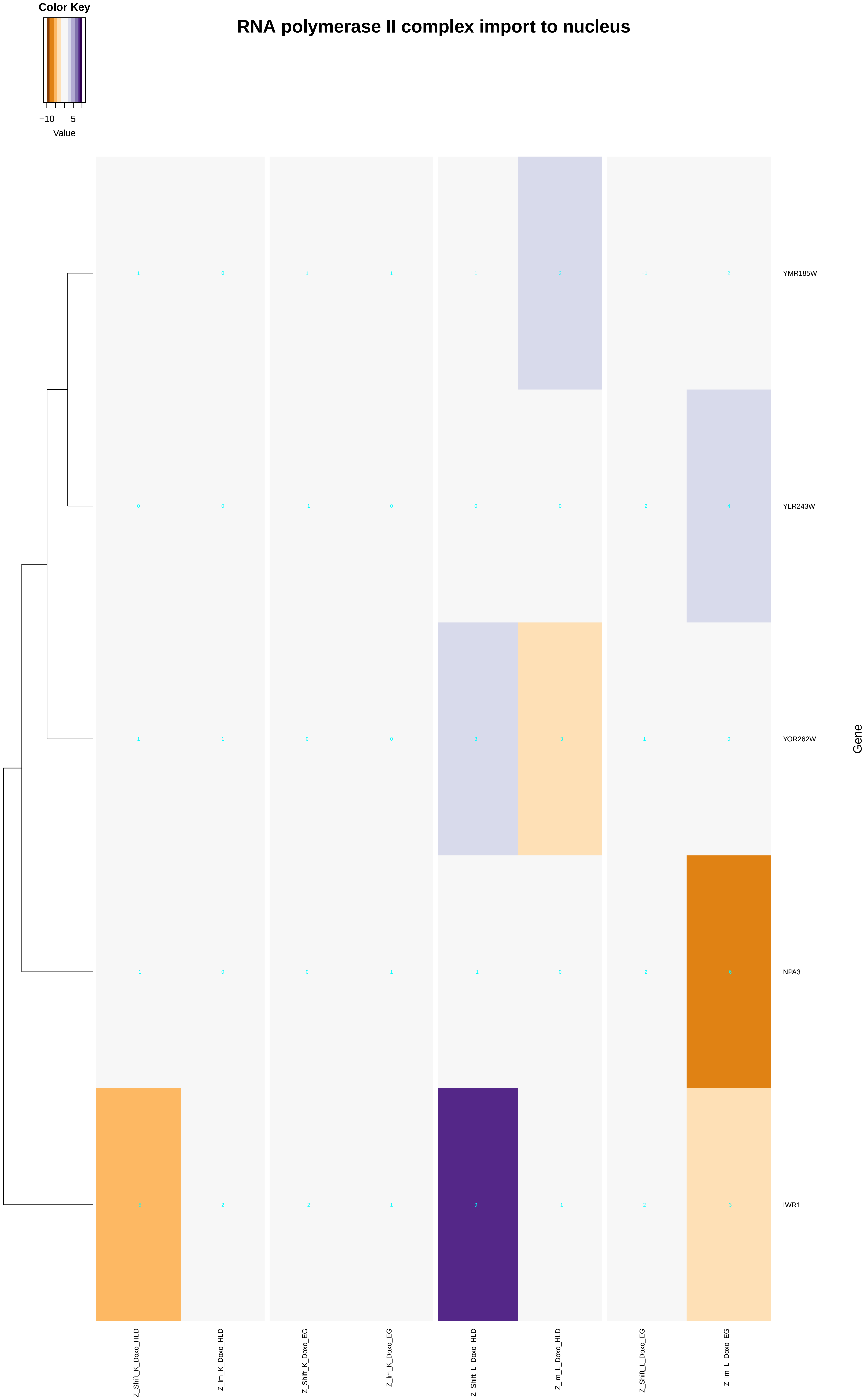

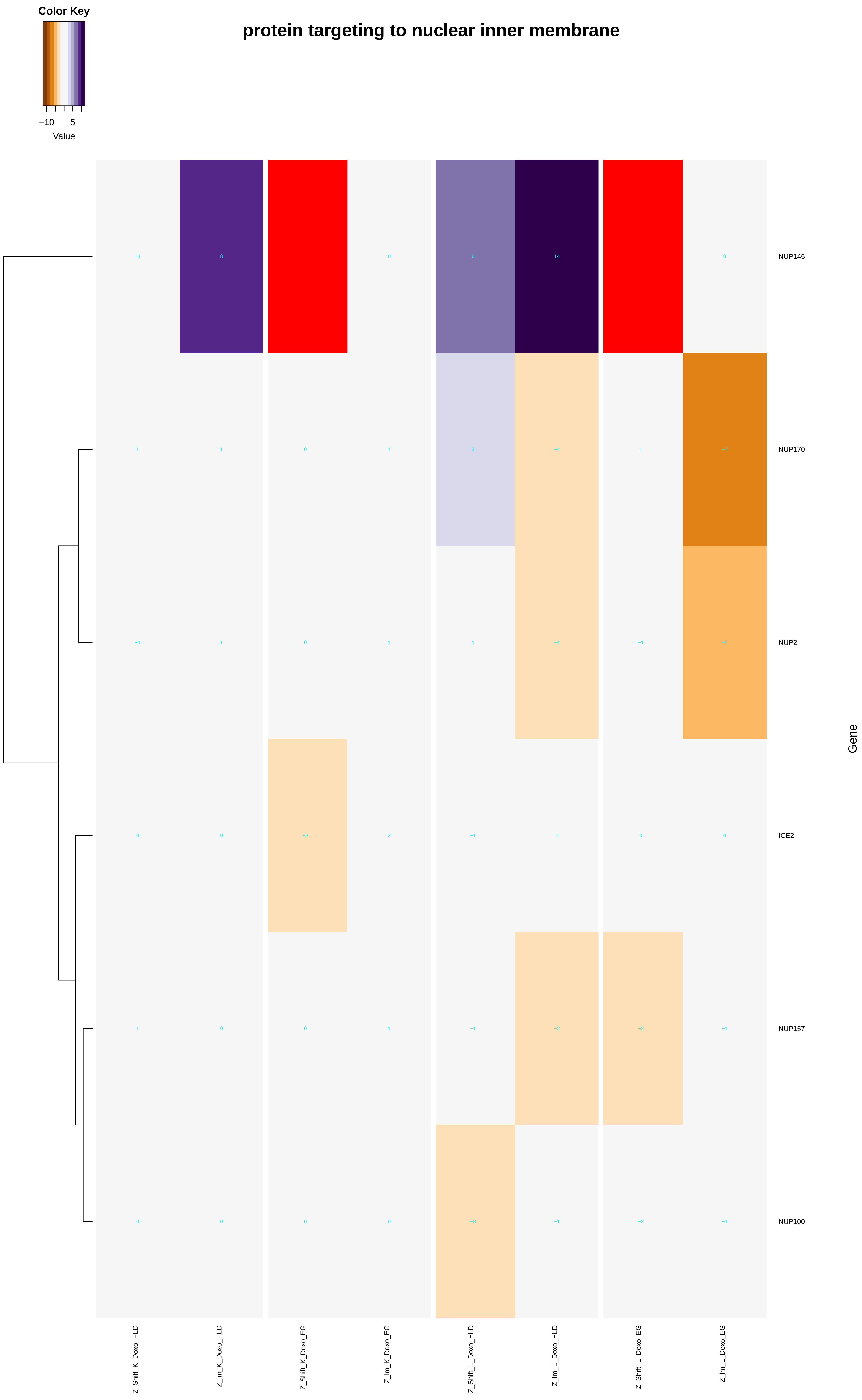
Color Key
−10
5
Value
protein targeting to nuclear inner membrane
NUP145
NUP170
NUP2
ICE2
NUP157
NUP100
Gene
Z_Shift_K_Doxo_HLD
Z_Im_K_Doxo_HLD
Z_Shift_K_Doxo_EG
Z_Im_K_Doxo_EG
Z_Shift_L_Doxo_HLD
Z_Im_L_Doxo_HLD
Z_Shift_L_Doxo_EG
Z_Im_L_Doxo_EG
